# Novel Genetic Tools for Diaminopimelic Acid Selection in Virulence Studies of *Yersinia pestis*

**DOI:** 10.1371/journal.pone.0017352

**Published:** 2011-03-02

**Authors:** David M. Bland, Nicholas A. Eisele, Lauren L. Keleher, Paul E. Anderson, Deborah M. Anderson

**Affiliations:** 1 Department of Veterinary Pathobiology, University of Missouri, Columbia, Missouri, United States of America; 2 Department of Molecular Microbiology and Immunology, University of Missouri, Columbia, Missouri, United States of America; Duke University, United States of America

## Abstract

Molecular studies of bacterial virulence are enhanced by expression of recombinant DNA during infection to allow complementation of mutants and expression of reporter proteins *in vivo*. For highly pathogenic bacteria, such as *Yersinia pestis*, these studies are currently limited because deliberate introduction of antibiotic resistance is restricted to those few which are not human treatment options. In this work, we report the development of alternatives to antibiotics as tools for host-pathogen research during *Yersinia pestis* infections focusing on the diaminopimelic acid (DAP) pathway, a requirement for cell wall synthesis in eubacteria. We generated a mutation in the *dapA-nlpB(dapX)* operon of *Yersinia pestis* KIM D27 and CO92 which eliminated the expression of both genes. The resulting strains were auxotrophic for diaminopimelic acid and this phenotype was complemented *in trans* by expressing *dapA* in single and multi-copy. *In vivo*, we found that plasmids derived from the p15a replicon were cured without selection, while selection for DAP enhanced stability without detectable loss of any of the three resident virulence plasmids. The *dapAX* mutation rendered *Y. pestis* avirulent in mouse models of bubonic and septicemic plague which could be complemented when *dapAX* was inserted in single or multi-copy, restoring development of disease that was indistinguishable from the wild type parent strain. We further identified a high level, constitutive promoter in *Y. pestis* that could be used to drive expression of fluorescent reporters in *dapAX* strains that had minimal impact to virulence in mouse models while enabling sensitive detection of bacteria during infection. Thus, diaminopimelic acid selection for single or multi-copy genetic systems in *Yersinia pestis* offers an improved alternative to antibiotics for *in vivo* studies that causes minimal disruption to virulence.

## Introduction

*Yersinia pestis* is the causative agent of plague and is a recently evolved pathogen [1], [2]. Due to its ability to undergo genetic flux from loss of genetic content and acquisition of DNA by horizontal transfer, *Y. pestis* evolved from a mild gastro-instestinal pathogen to one that rapidly induces high titer sepsis in mammals in order to promote its transmission and environmental survival in fleas [3]. Many biovars of *Y. pestis* exist, varying between one another by significant changes including plasmid acquisition, while even within biovars, strains differ due to numerous point mutations, often in non-coding sequences [4], [5]. Isolation of multi-antibiotic resistant *Y. pestis* from human plague patients has been reported in two independent cases, both of which were due to the acquisition of different multi-drug resistant plasmids highlighting a potential public health concern for the evolution of drug resistant plague [6], [7], [8]. This, combined with its hypervirulence in humans and mammals, stable maintenance in the environment between outbreaks, and the potential for rapid spread among humans, makes *Yersinia pestis* a potential reemerging threat to public health.

Heightened concern over highly pathogenic microbes such as *Yersinia pestis* has led to a surge in plague investigations, from basic mechanisms of pathogenesis to the development of novel vaccines and therapeutics. Yet, currently available gene expression and gene knockout tools used for attenuated *Yersinia* strains rely on the introduction of antibiotic resistance which is restricted in the virulent isolates, thereby limiting the potential output of this surge in research activity. In this work, we addressed this shortfall and report the adaptation of standard genetic tools for metabolic rather than antibiotic selection.

Biosynthesis of lysine has become an increasingly used anti-bacterial target as it provides essential protein (lysine) and cell wall (meso-diaminopimelic acid) components, thereby inhibiting bacterial growth by two mechanisms [9]. Mammals are unable to synthesize lysine and lack diaminopimelic acid, therefore the presence of a functional lysine biosynthetic pathway is essential for bacterial growth in mammalian hosts. Like antibiotics, this property has been explored as a mechanism for selection of bacteria carrying recombinant plasmids during infection. For example, *Salmonella typhimurium* lacking *asdA* (aspartate dehydrogenase) is unable to synthesize diaminopimelic acid and therefore is avirulent in a mouse model of disease [10], [11]. Growth of this mutant is dependent on exogenous diaminopimelic acid or on the plasmid expression of *asdA* allowing for its selection *in vivo*. In *E. coli*, deletion of *dapA, B, C, D* and *E* confer diaminopimelic acid auxotrophy that can be used to select for recombinant DNA [12]. Selection systems involving *dapB* (dihydropicolinic acid reductase) have been reported for other Gram negative pathogens such as *Burkholderia pseudomallei*, thus it appears there are multiple genetic targets to block this highly conserved metabolic pathway [13], [14].

In this work, we explored the utility of diaminopimelic acid selection in *Yersinia pestis* for single and multi-copy expression of recombinant DNA. In *Y. pestis*, the genes encoding *dapB, C, D* and *E* are duplicated with two copies of each present in the chromosome [15]. However the gene encoding *dapA*, a dihydropicolinic acid synthetase, is present in another chromosomal location, found in single copy, and is therefore predicted to be necessary for an early step of the pathway for biosynthesis of diaminopimelic acid. In *Y. pestis*, as well as many other bacteria, *dapA* is annotated as the first gene of an operon that includes *nlpB/dapX*, an outer membrane lipoprotein that is not essential for growth [16]. Here we show that null mutation of the *dapAX* operon results in diaminopimelic acid (DAP) dependent growth and an avirulent phenotype in mouse models of plague. Growth without DAP could be restored by supplying *Y. pestis dapA* in single or multiple copies and retention of plasmids could be achieved *in vivo* during murine infection. Complementation of the *dapAX* mutation *in vivo* required the introduction of both genes *in trans*, either in single or multiple copy, and this restored the development of plague to near wild type levels. We used this system to generate a sensitive DAP-selectable fluorescent expression system that enables identification of bacteria during infection. Together the data demonstrate the utility of the diaminopimelic acid biosynthetic pathway as an improved alternative to antibiotic selection for expression of recombinant DNA during experimental models of plague.

## Materials and Methods

### Bacterial strains and growth conditions

*Y. pestis* strains used in this study are listed in Table 1 and *E. coli* strains are listed in Supplemental Table S1. All strains used were taken from frozen stocks and streaked for isolation onto heart infusion agar (HIA) plates. The plates used for *Y. pestis* CO92 were supplemented with 0.005% Congo Red and 0.2% galactose to identify bacteria that retain the pigmentation locus [17]. For bubonic plague challenge, a single red pigmented colony was used to inoculate heart infusion broth (HIB) and grown 18–24 hrs at 26°C, 120 rpm. All handling of samples containing live *Y. pestis* CO92 was performed in a select agent authorized BSL3 facility under protocols approved by the University of Missouri Institutional Biosafety Committee. *Y. pestis* KIM D27, a non-pigmented strain originally isolated by Robert Brubaker, was routinely grown fresh from frozen stock on HIA, followed by aerobic growth at 26°C in HIB overnight prior to use in experiments [18]. Where indicated, ampicillin (100 µg/ml) was added to media for selection of plasmids. For growth of *dapA* mutant *Y. pestis*, 400 µg/ml diaminopimelic acid (DAP) (Sigma Aldrich, St Louis, MO) was added to liquid or agar media.

**Table 1:** Bacterial strains used in this study.

*Y. pestis* Strains	Key Properties	Reference
KIM D27	Pgm^-^ Lcr^+^; KIM 5 derivative	[18]
KIMD27-1003	KIM D27; Missing *dapA* promoter and entire *dapA* ORF, generated with pCVD442-dapAX	This Study
KIMD27-1011	KIM D27*dapAXatt*Tn7*::dapA*; *dapA* transposition downstream of glmS of the KIMD27-1003 parent strain	This Study
KIMD27-1012	KIM D27*dapAXatt*Tn7*::dapAX*; *dapAX* transposition into the attTn7 site of the KIMD27-1003 parent strain	This Study
KIMD27-1013	KIM D27*dapAXatt*Tn7*::dapAX DsRed*; *dapAX* and *cysZ-DsRed* transposition into the *att*Tn7 site of the KIMD27-1003 parent	This Study
KIMD27-1014	KIM *D27dapAXatt*Tn7*::dapAX Tomato*; *dapAX* and *cysZ-Tomato* transposition into the *att*Tn7 site of the KIMD27-1003 parent	This Study
CO92	Pgm^+^ Lcr^+^	[33]
CO92-1008	CO92; Missing *dapA* promoter and entire *dapA* ORF, generated with pCVD442-dapAX	This Study
CO92-1009	CO92*dapAXatt*Tn7*::dapAX*; *dapAX* transposition into the *att*Tn7 site of the CO92-1008 parent strain	This Study
CO92-1010	CO92*dapAXatt*Tn7*::dapAX DsRed*; *dapAX* and *cysZ-DsRed* transposition into the *att*Tn7 site of the CO92-1008 parent strain	This Study
CO92-1011	CO92*dapAXatt*Tn7*::dapAX Tomato*; *dapAX* and *cysZ-Tomato* transposition into the *att*Tn7 site of the CO92-1008 parent strain	This Study

*E. coli* DH5α and JM109 served as cloning strains for construction of recombinant pACYC177 and pBR322 (New England Biolabs, Ipswich, MA) based plasmids [19], [20]. *E. coli* S17-1λpir [21] served as cloning strain for Ori-R6K based plasmids, including the suicide vector and the mini-Tn7 vectors [22], [23]. *E. coli* strains were grown in LB media for propagation. For cloning purposes, ampicillin (100 µg/ml) was added to the media for selection.

### Plasmids and dapAX complementation

Plasmids and primers used or developed in this study are listed in Supporting Information, Tables S1, S2 and S3. *Yersinia pestis dapA* and *dapAX* were amplified from *Y. pestis* KIM D27 by PCR. pACYC177 was modified by replacement of the kanamycin resistance gene with that of *dapA* and its endogenous promoter using restriction sites HindIII and SmaI. The resulting plasmid no longer conferred kanamycin resistance but still retained ampicillin resistance and was used for complementation studies. The suicide vector, pCVD442 *dapA*, was constructed by amplifying 1,000 bp upstream of the *dapA* promoter and 1,000 bp downstream of the *dapA* stop codon [22], [24]. These DNA fragments were amplified by PCR and ligated into the XbaI and SphI sites of pCVD442 using EcoRI as a linker between upstream and downstream DNA segments. Restriction enzymes and T4 DNA ligase were purchased from New England Biolabs (Ipswich, MA). Promoterless DsRed was amplified from pDsRed Monomer (Clontech, Mountain View, CA) and cloned into pBR322 for use in the promoter trap screen. For Tn7 transposition, *dapA* or *dapAX* was amplified by PCR and cloned into the SmaI and SpeI sites of pUC18R6KT mini-Tn7 [23].

### Animals

This study was carried out in strict accordance with the recommendations in the Guide for the Care and Use of Laboratory Animals of the National Institutes of Health and was approved by the Animal Care and Use Committee of the University of Missouri. All efforts were made to minimize suffering of the animals. Male and female BALB/c or C57BL/6 mice, 6–8 weeks old, were either purchased from Charles River Laboratories (Wilmington, MA) or were bred and raised in barrier facilities at the University of Missouri. During bubonic plague challenge, mice were maintained in select agent approved containment facilities at the University of Missouri. All infected mice were monitored regularly by daily weighing and assignment of health scores. Animals that survived to the end of the 14 day observation period or were identified as moribund (defined by severe ataxia) were euthanized by CO_2_ asphyxiation followed by bilateral pneumothorax, methods approved by the American Veterinary Medical Association Guidelines on Euthanasia.

### Plague challenge

For bubonic plague, *Y. pestis* CO92, grown as described above, was diluted in sterile PBS to the indicated dose just prior to use for challenge experiments. Bacteria were delivered in 100 µl volume by subcutaneous injection in BALB/c mice (LD50 = 1 CFU) [25]. Actual dose and retention of the pigmentation locus were determined by plating in triplicate on HIA with congo red. For intravenous challenge involving non-pigmented *Y. pestis* KIM D27, bacteria were diluted in sterile PBS and delivered by tail vein injection of 100 µl (LD50 = 100 CFU) into BALB/c or C57BL/6 mice [26]. For intranasal challenge involving non-pigmented *Y. pestis* KIM D27, 20 µl of bacteria grown and diluted as described above, was delivered to BALB/c mice that were pre-treated by intraperitoneal injection of 500 µg Fe^+2^ (FeCl_2_). All animals subcutaneously or intranasally infected with *Y. pestis* were first lightly anesthetized by isoflurane inhalation. Animals were observed for recovery from anesthesia following the procedure and returned to housing. LD50 determination was performed by the method of Reed and Muench [27].

### Competitive Index

This method was performed as previously described [28]. Wild type *Y. pestis* KIM D27 with or without recombinant pACYC*dapA* were combined in a 1∶1 ratio (doses ranging from 1,000 to 13,000 CFU each strain) and injected intravenously into BALB/c or C57BL/6 mice in a 100 µl volume. Four days post infection, mice were euthanized, and spleens were harvested, homogenized in PBS and plated in duplicate on HIA (all bacteria) and HIA + amp (plasmid-bearing bacteria). To calculate plasmid loss, bacterial colony forming units (CFU) recovered without amp selection were subtracted from the CFU recovered with amp selection and percentages of each found in the spleen were determined. The Competitive Index (C.I.) is defined as: % amp^r^ recovered/% amp^r^ input. For statistical analysis, the proportions of amp^r^ to total CFU recovered was compared with the ratio of amp^r^ to total CFU in the inoculum.

### *Yersinia* promoter trap screen

Primers with abutted restriction sites were used to amplify the open reading frame of DsRed-Monomer (Clontech, Mountain View, CA) which was subsequently ligated into pBR322 (New England Biolabs, Ipswich, MA) in place of the tetracycline resistance gene. *Y. pestis* KIM D27 genomic DNA was digested with RsaI and 100–1,50 bp DNA fragments were ligated directly upstream of DsRed. Colonies were screened in *E. coli* DH5α for DsRed expression, and those that gave the strongest signal were transformed into *Y. pestis* KIM D27 and checked for DsRed fluorescence. One plasmid from this screen, pRsaI-2.1, was further characterized by sequence analysis, and then sub-cloned into pDB2 (pUC18R6KT + *dapAX*). The resulting plasmid was then used for transposition into *Y. pestis* KIM D27- 1003 (*dapAX*) to insert DsRed and *dapAX* into the chromosome. The *cysZK* promoter-containing fragment was amplified from *Y. pestis* KIM D27 with primers which have abutted XhoI and EcoRV sites. The *lac* promoter of ptdTomato (Clontech, Mountain View, CA) was replaced with the *cysZK* promoter fragment creating pNE160. For Tn7 transposition, the *P_cys_*Tomato fragment was digested from pNE160 with XhoI and EcoRI, and ligated into the XhoI and EcoRI sites of pDB2.

*DsRed expression assay.* Single colonies of the indicated strains were used to inoculate HIB and grown overnight at 26°C or 37°C; 1 mL of culture was removed and pelleted at 6000 rpm for 5 minutes then resuspended in 4% paraformaldahyde and incubated for 20 minutes. Samples were then washed once and resuspended in 1 mL PBS. Samples were analyzed on a FluoStar Omega plate reader for absorbance (600 nm) and DsRed fluorescence (544/590 nm). Measured fluorescent values were normalized to cell number.

### Macrophage assay

Macrophages were prepared as previously described [29]. Briefly, 1×10^6^ biotinylated macrophages were plated in DMEM supplemented with 5% FBS and infected with the indicated strains at an MOI of 10 for 30 min. Cells were fixed with 4% paraformaldahyde then stained with DAPI and streptavidin conjugated to Alexa Fluor 488 (Invitrogen, Carlsbad, CA) and analyzed by confocal microscopy.

### Statistical Analyses

Data from the competitive index were tested for difference from a given proportion using prop test from R [30]. Briefly, the proportion of amp^r^ to total CFU in the recovery was tested for a difference from the proportion of amp^r^ to total CFU in the inoculums using alpha = 0.05. Survival data was evaluated by a modified Gehan-Breslow-Wilcoxon Test [31]. DsRed expression data were evaluated by one way ANOVA. Significance was concluded for p<0.05.

## Results

### Deletion of dapAX in *Y. pestis* results in DAP auxotrophy

**Figure 1:**
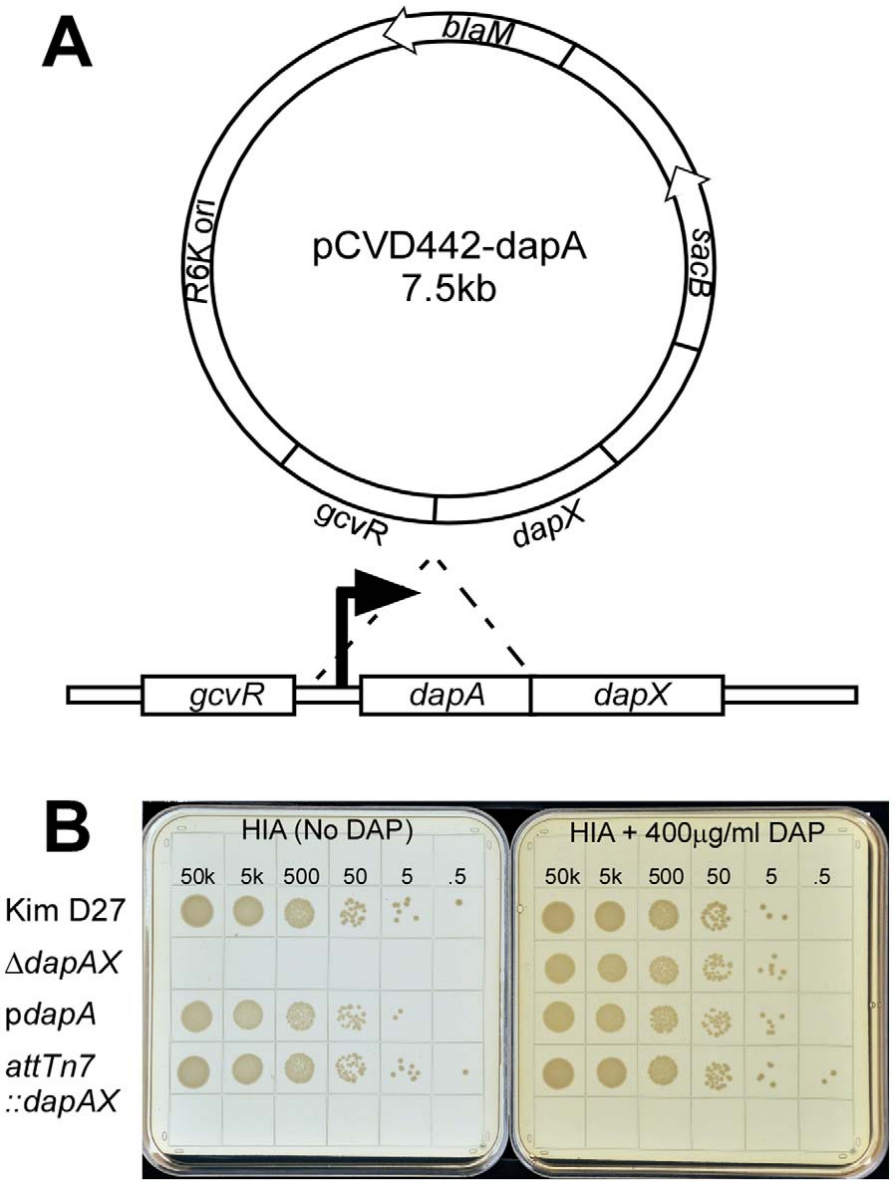
Construction of the *dapAX* mutation results in DAP dependent growth. The *dapA* promoter and open reading frame were deleted by homologous recombination using the plasmid pCVD442 (A). Overnight cultures of the indicated strains (the *dapAX* mutant supplemented with 400 µg/ml DAP) were serially diluted 10 fold in HIB (no DAP) and plated on HIA with or without DAP and incubated at 26°C for 48 hrs (B).

Diaminopimelic acid (DAP) is a component of the cell wall that provides cross linking of peptidoglycan in many Gram negative bacteria including *Yersinia pestis*. Previous work showed that disruption of the metabolic pathway for biosynthesis of DAP renders *E. coli* unable to grow in media lacking diaminopimelic acid [12]. Thus, standard laboratory media such as heart infusion agar, blood base agar and Luria agar cannot support growth of mutants lacking essential genes of the diaminopimelic acid biosynthetic pathway. A search of the *Y. pestis* genome revealed that many genes are duplicated, but one gene required for an early step in the biosynthetic pathway, dipicolinate synthetase or *dapA*, was present in single copy [15]. We therefore generated a suicide vector designed to delete the promoter and open reading frame of *dapA* in *Yersinia pestis*, which is predicted to delete the expression of two genes, *dapA* and *nlpB* (*dapX*), likely present in an operon (Figure 1A). Homologous recombination of the deletion construct was introduced by pCVD442 into the wild type, non-pigmented *Y. pestis* strain KIM D27 and resulted in a mutant strain that was unable to grow on plates without DAP supplementation or expression of *dapA in trans* (Figure 1B, data not shown). Deletion of *dapA* was confirmed by PCR, and the absence of *dapA* and *dapX* mRNA was observed by reverse transcriptase PCR of mRNA purified from stationary phase cultures (data not shown).

**Figure 2:**
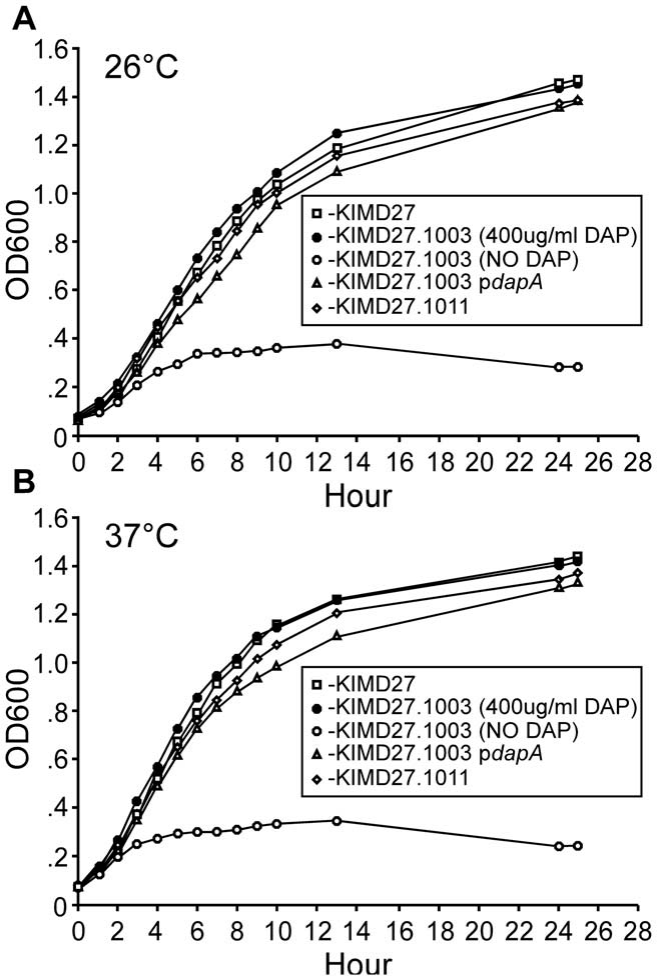
DAP independent growth is restored by expression of *dapA* in single or multi copy. Wild type KIM D27, and isogenic strains KIM D27-1003, KIM D27-1003p*dapA*, or KIM D27-1011 were grown overnight in HIB at 26°C, then diluted to an OD600 of .075 and grown for 25 hrs at 26°C (A) or 37°C (B) with shaking at 130 rpm, monitoring OD600 as indicated. KIM D27-1003 strain with no DAP supplementation was washed 3X in sterile PBS to remove excess DAP from the overnight culture. Data are representative of 3 independent experiments.

*DAP independent growth is restored by expression of dapA in trans*. We next characterized the *Y. pestis* KIM D27 *dapAX* strain for growth characteristics in laboratory media with and without DAP. The *dapAX* strain was unable to grow in broth media without DAP, either at 26°C or 37°C and this was restored by supplying the wild type *dapA* gene in either single or multiple copies (Figure 2). However, the *dapAX* mutant grew normally when DAP was added to the culture media. Following removal of DAP from the media, viability of the *dapAX* mutant sharply declined 6 hrs later indicating depletion of DAP is rapidly lethal to the bacteria (data not shown). Together the data suggest that the absence of DapA confers dependency on supplemental diaminopimelic acid for growth.

**Figure 3:**
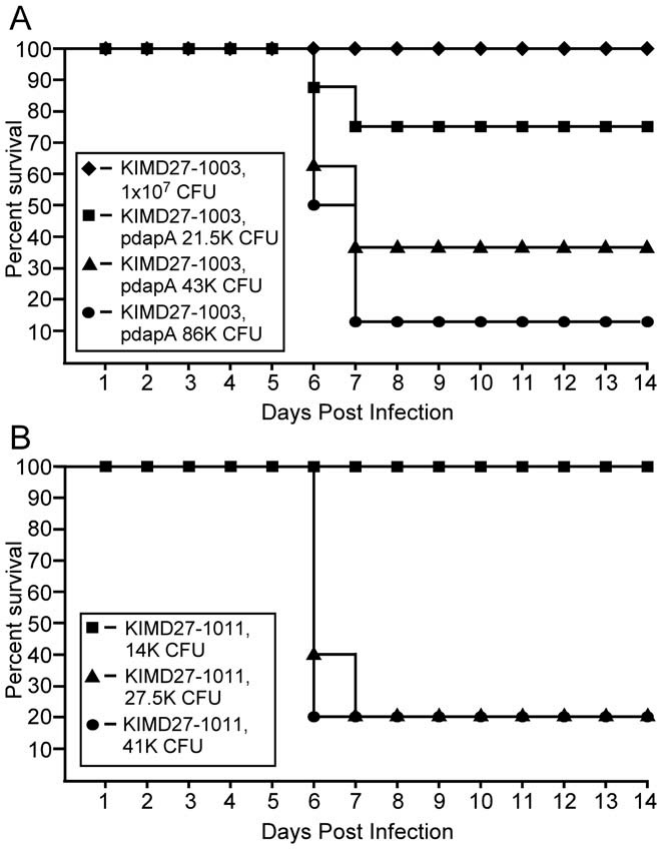
Expression of *dapA* on a multi-copy plasmid partially restores virulence. *Y. pestis* KIM D27-1003 (*dapAX*) either with or without p*dapA* (A), or with *dapA* inserted into the *att*Tn7 site of *Y. pestis* KIM D27 (KIM D27-1011) (B) were grown overnight in HIB either with or without 100 µg/ml ampicillin, then diluted in sterile PBS to the indicated doses. 100 µl was injected intravenously into the tail vein of BALB/c mice (n = 8 per group for A; n = 5 per group for B). Survival was monitored for 14 days. The observed 50% lethal dose (LD50) was calculated as 30,409 (p*dapA*) and 20,804 (KIM D27-1011) by the method of Reed and Muench [27].

We tested for diaminopimelic acid selection during a mouse model of septicemic plague. In this model, pACYC *dapA* was introduced by electroporation into *Y. pestis* KIM D27-1003 or *dapA* was inserted in single copy into the chromosome of the *dapAX* mutant using Tn7 transposition, and the resulting strains were used to infect BALB/c mice by intravenous injection [23], [32]. Whereas the *dapAX* mutant was avirulent, with >10^6^ fold increase in dose required for a lethal infection in this model, both single and multiple copy complementation with *dapA* led to substantial, but not complete, restoration of virulence, with calculated LD50s of 30,409 CFU or 20,804 CFU, respectively, roughly 200–300 fold decrease in virulence compared to wild type *Y. pestis* KIM D27 (Figure 3).

### Diaminopimelic acid selection is functional *in vivo* in mouse models of plague

To investigate whether or not the *dapA* plasmid was stably maintained *in vivo*, we isolated bacteria from the spleens of BALB/c mice infected with 20,000 CFU of *Y. pestis* KIM D27-1003pACYC-*dapA* on day 4 post-infection, when each mouse showed signs of acute disease. Colonies isolated from these spleens were tested by PCR to verify the presence of all three *Y. pestis* virulence plasmids in addition to the plasmid expressing *dapA*. PCR analysis of 81 colonies from each mouse verified a high degree of retention of all three virulence plasmids as no plasmid loss was seen (Table 2). These results strongly suggest that p15a plasmids can be selected *in vivo* without loss of resident *Y. pestis* virulence plasmids. However, since the pACYC-*dapA* strain was unable to fully restore virulence to wild type levels, we sought to further characterize the impact of p15a plasmids on the virulence of wild type *Y. pestis*.

**Table 2:** DAP selection *in vivo* does not cause instability of resident virulence plasmids.

Virulence plasmid	Gene	PCR Positive Colonies	% Retention
**pCD1**	*lcrH*	81/81	100%
	*yopB*	81/81	100%
**pPCP1**	*pla*	81/81	100%
	*pst*	81/81	100%
**pMT1**	*caf1*	81/81	100%
	*ymt*	81/81	100%
**pACYC** ***dapA***	*bla*	81/81	100%

### Diaminopimelic acid selection is necessary for plasmid retention *in vivo*

**Figure 4:**
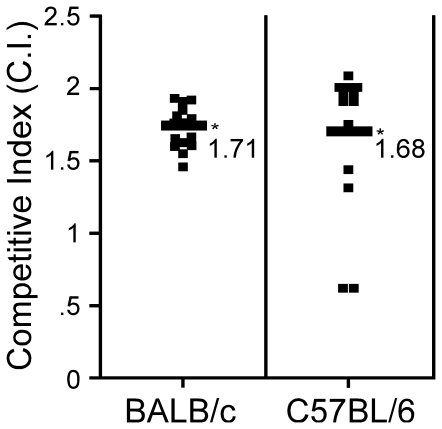
*pACYC177* imposes a biochemical burden on *Y. pestis in vivo*. *Y. pestis* KIM D27 with or without p*dapA* was grown overnight in HIB with or without, respectively, ampicillin. An approximately 1∶1 ratio of each strain was mixed and delivered by intravenous injection into the tail vein of BALB/c (A) or C57BL/6 (B) mice. On day 4 post-infection, when many of the mice were moribund, spleens were harvested and bacterial titer was determined for strains with and without plasmid by plating serial dilutions on HIA and HIA+amp. The Competitive Index (C.I.) is defined as the ratio of recovered bacteria from mouse spleens divided by the ratio in the inoculum. Scores less than one indicate the plasmid-bearing strain was less fit than its counterpart within an individual mouse. After no significant difference between experiments were detected, data were pooled from 3 independent experiments with groups of 4–5 mice, and a total of 15 (BALB/c) and 13 (C57BL/6), respectively, were analyzed. Data were tested for difference of proportion using R giving p<0.0001.

To understand the effects of p15a plasmids on the virulence of *Y. pestis*, we performed a competition experiment to determine if pACYC-*dapA* impaired growth *in vivo*. Towards this end, BALB/c or C57BL/6 mice were challenged by intravenous injection of 10^3^–10^4^ CFU wild type *Y. pestis* KIM D27 mixed in a 1∶1 ratio with wild type bacteria expressing pACYC *dapA*. Following 4 days post-infection, mice were euthanized and bacteria in the spleens were enumerated. KIM D27 cells harboring pACYC *dapA* compared to those without plasmid were identified by plating bacteria on media with and without ampicillin. The percentages of plasmid carrying strain recovered from the spleen were compared to the input values to calculate the competitive index (CI) (Figure 4). The difference in proportion between input and recovery between infections in BALB/c and C57BL/6 mice was then evaluated for statistical significance. Both strains of mice yielded similar results, and in nearly all mice, bacteria carrying the plasmid decreased in proportion after infection (p<0.001) and the corresponding CI was typically greater than 1 for bacteria without plasmid. Together, these results suggest that carrying an additional plasmid, though it may not cause instability of other virulence plasmids, imposes a biochemical burden that either retards bacterial growth *in vivo* or causes it to be subject to curing during the infection.

We therefore also measured stability of pACYC177 and pACYC*dapA* during *Y. pestis* KIM D27 infection of BALB/c mice without selection. Bacteria harvested from the spleen on day 4 post-infection were monitored for loss of ampicillin resistance by plating initially on HIA, then patching colonies onto plates with and without ampicllin. Results showed plasmid loss for both strains ranging from 1–5% with higher loss for the larger plasmid containing *Y. pestis dapA* (Table 3). Together the data indicate that p15a plasmids are cured during infection and thus may be incompatible with one or more virulence plasmids.

**Table 3:** p15a plasmid loss with no selection detected in spleens recovered from moribund mice.

Mouse	Total CFU^a^ (from spleen)	Amp^S^ CFU	Plasmid Loss^b^
1- *pdapA*	100	5	5%
2- *pdapA*	100	2	2%
3- *pdapA*	80	3	3.75%
**Combined**	280	10	3.5%
1- *pACYC-177*	100	1	1%
2- *pACYC-177*	60	1	1.66%
3- *pACYC-177*	97	1	1.03%
**Combined**	257	3	1.17%

a: CFU: Colony forming units of *Y. pestis* KIM D27.

b: Amp^s^/total CFU×100.

### Single copy complementation of *dapAX* restores virulence

**Figure 5:**
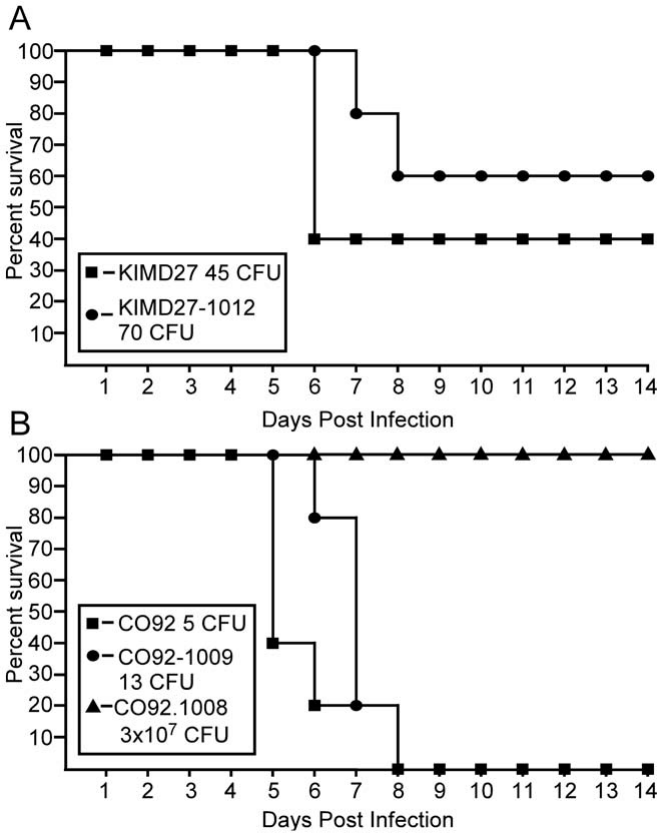
Complementation of the *dapAX* operon by chromosomal insertion restores virulence. (A) *Y. pestis* KIM D27 and KIM D27-1012 (*dapAX att*Tn7*::dapAX*) were grown overnight, diluted to the indicated dose in sterile PBS and delivered by intravenous injection into the tail vein of BALB/c mice. (B) *Y. pestis* CO92, CO92-1008 (*dapAX*), and CO92-1009 (*dapAX attTn7*::*dapAX*) were grown overnight, diluted to the indicated dose in sterile PBS and delivered by subcutaneous injection into the left hind limb of BALB/c mice (n = 5 for all groups). Survival was monitored over 14 days for both models. No significant difference in survival was detected between wild type and *dapAX* complemented strains (p = 0.22 for KIM D27; p = 0.10 for CO92) using the Gehan-Breslow-Wilcoxon test.

Because of the biochemical burden imposed by plasmids, we tested whether the virulence defect that remains in KIM D27*att*Tn7*::dapA* was caused by the lack of *dapX*/*nlpB*, a non-essential gene with no previously known role in virulence. The *dapAX* operon was cloned into the multi-cloning site of pUC18R6KT which is flanked by *att*Tn7 transposition sites. The resulting plasmid, pDB2, and the helper plasmid encoding the transposase complex, pTNS2, were electroporated into *Y. pestis* KIM D27-1003, and selected on HIA (no DAP). The complemented strain was verified by PCR to carry *dapAX* downstream of *glmS* rather than its original location on the chromosome (data not shown). To test complementation *in vivo*, we infected BALB/c mice with *Y. pestis* KIM D27*dapAX att*Tn7::*dapAX* by intravenous injection and challenged with a dose equivalent to 1 LD50 of the wild type parent strain. High challenge doses of either wild type or the *dapAX* complemented strain resulted in 100% lethality (data not shown). Moreover, lethality was also similar at low challenge dose (60% for wild type KIM D27 and 40% for the single copy *dapAX* complemented strain) suggesting single copy expression of *dapAX* is sufficient to restore virulence to near wild type levels (Figure 5A).

**Figure 6:**
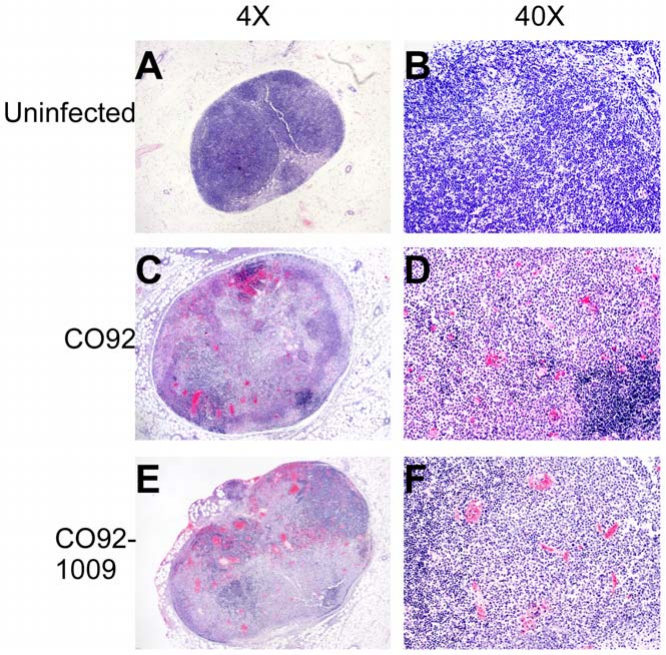
Development of fulminant bubonic plague is restored by *att*Tn7::*dapAX*. *Y. pestis* CO92 and CO92-1009 (*dapAX att*Tn7*::dapAX*) were grown overnight at 26°C, diluted in sterile PBS to the indicated doses and delivered to BALB/c mice by subcutaneous injection. On day 4 post-infection, the left inguinal lymph node was removed, fixed in formalin, sectioned and stained with hematoxylin and eosin (H&E). (A–B) Uninfected; (C–D) CO92; (E–F) CO92-1009. Images are representative of tissues harvested from 5 mice in each group.

We also tested whether DAP selection functioned in the fully virulent *Orientalis Y. pestis* strain CO92. The *dapAX* mutation was generated by deletion of the promoter and open reading frame for *dapA* using pCVD442 and homologous recombination as described above. The resulting strain was unable to grow on media without supplemental DAP (data not shown). The deletion was confirmed by PCR as well as retention of all three virulence plasmids and the pigmentation locus (data not shown). Introduction of *dapAX* in single copy using the mini-Tn7 transposon restored growth in the absence of supplemental DAP. The *Y. pestis* CO92 *dapAX* mutant and *dapAX att*Tn7::*dapAX* strains were then used to challenge BALB/c mice by subcutaneous injection in a bubonic plague model. In this model, insertion of *dapAX* by Tn7 transposition also appeared to fully complement virulence with 100% lethality caused by less than 15 CFU of either wild type or complemented strain whereas *dapA* alone did not fully restore virulence (Figure 5B, unpublished observations) [33]. Histopathology of moribund mice indicated development of bubonic plague as lymph nodes taken from subcutaneously challenged mice on day 4 post-infection showed severe hemorrhage and necrosis similar to wild type (Figure 6). Thus, with DAP as a selection for Tn7 insertion of genes in single copy, virulence could be restored indicating no significant impact on pathogenesis.

### DAP selectable system for single copy detection of fluorescence *in vivo*

To exploit DAP selection for virulence studies, we screened for constitutively active *Yersinia* promoters that could drive high level expression of the fluorescent protein DsRed permitting detection by confocal microscopy in single copy. Towards this end, a library of *Y. pestis* KIM D27 DNA fragments (100–1,500 bp) fused to a promoterless DsRed plasmid was generated in *E. coli*. Colonies were screened for expression of DsRed and those with the strongest signal were then tested for activity in *Y. pestis*. The strongest isolate, RsaI-2.1, could be identified on agar media as a red colony in both *E. coli* and *Yersinia* (data not shown). The insert was characterized by sequencing, revealing the presence of the *cysZK* promoter and first 178 codons of its open reading frame fused in frame to DsRed. We then cloned this *cysZK* promoter element from *Y. pestis* and generated Tn7 constructs for the red fluorescing proteins DsRed and tdTomato.

**Figure 7:**
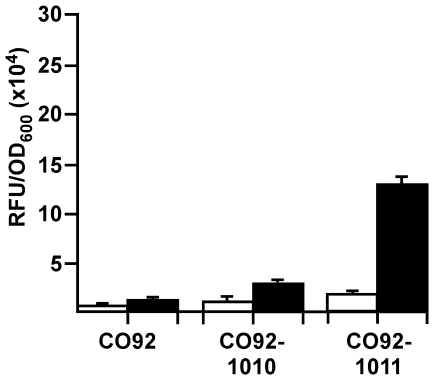
The *cysZK* promoter supports high level expression of fluorescence in single copy in *Y. pestis*. *Y. pestis* CO92 strains were grown overnight in HIB at 26°C (open bars) or 37°C (closed bars) and then analyzed on a 96-well plate. Relative fluorescent units (RFU) were measured on a plate reader at an excitation/emission spectra of 544/590 nm. Each value was normalized to the OD600 of the sample. To facilitate removal from the BSL-3 laboratory, 1 mL of culture was removed, fixed in 4% paraformaldahyde then resuspended in PBS. Error bars represent the standard error of the mean between three distinct overnight cultures.

P*_cys_*-DsRed and P*_cys_*-Tomato reporters were introduced into *Y. pestis* CO92*dapAX* by Tn7 transposition and selected by growth on HIA without DAP supplementation. The resulting strains were confirmed by PCR (data not shown) and expression of DsRed or Tomato was monitored in overnight cultures incubated at 26°C or 37°C in HIB. The results showed strong expression of DsRed, and even stronger red fluorescence was observed in the strain expressing Tomato (Figure 7). Remarkably, expression of DsRed or Tomato in this system did not have a significant impact on virulence compared to complementation with *dapAX* alone in an intranasal model of septicemic plague, as challenge doses of approximately 50X LD50 caused similar lethality (Table 4) [34]. Likewise, expression of DsRed or Tomato in fully virulent *Y. pestis* CO92 caused lethality similar as wild type when challenged with 50X LD50 in a bubonic plague model.

**Table 4:** High level, constitutive expression of DsRed or Tomato causes minimal disruption to virulence.

Strain	Percent Survival	Challenge Dose
KIM D27	16.7 (1/6)	5.6×10^5 ^ a
KIM D27pRsaI2.1	0 (0/6)	5.3×10^5 ^ a
KIM D27-1014 (*dapAXatt*Tn7*::Tomato*)	0 (0/9)	5.7×10^5 ^ a
CO92	0 (0/8)	58^b^
CO92 -1010 (*dapAX att*Tn7*::DsRed)*	12.5 (1/8)	66^b^
CO92-1011 (*dapAX att*Tn7*::Tomato)*	12.5 (1/8)	60^b^

a: Challenge by intranasal instillation; mice pre-treated with 500 µg Fe^+2^ just prior to challenge; dose is equivalent to 50X LD50 for wild type KIM D27.

b: Challenge by subcutaneous injection; dose is equivalent to 50X LD50 for wild type CO92.

**Figure 8:**
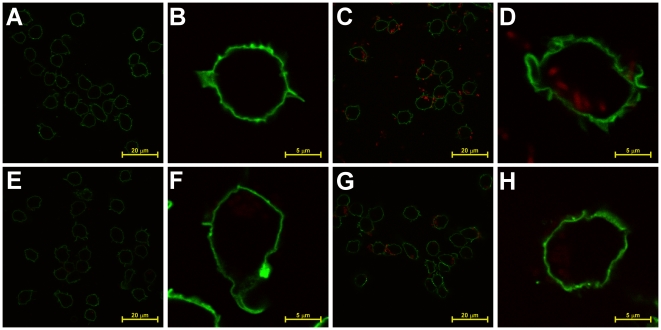
Detection of intracellular and extracellular bacteria by microscopy of single copy expression of *P_cys_Tomato.* (A–B) *Y. pestis* KIM D27, (C–D) pRsaI-2.1, (E–F) KIM D27-1013 (*dapAX att*Tn7*::dapAX P_cys_DsRed*), or (G–H) KIM D27-1014 (*att*Tn7*::dapAX cys-Tomato*) were grown overnight in HIB at 26°C, diluted 1∶15 in HIB and grown for 2 hours, and then used to infect biotinylated RAW 264.7 macrophage-like cells at an MOI of 10 for 30 minutes. Cells were then fixed and stained with streptavidin conjugated to AlexaFluor 488 to indentify the host cell membrane. Samples were analyzed by laser scanning confocal microscopy.

Next, we tested fluorescence expression during macrophage infections. *Y. pestis* KIM D27pRsaI-2.1 (multi-copy DsRed), KIM D27-1013 (single copy DsRed) or KIM D27-1014 (single copy Tomato) were grown at 26°C overnight, diluted in sterile PBS and added to biotin labeled RAW 264.7 macrophage-like cells. Infection was initiated by centrifugation, and after 30 min, cells were fixed and labeled with streptavidin-Alexa Fluor 488 to enable fluorescent detection of macrophages, then examined by confocal microscopy (Figure 8). Expression of DsRed from this plasmid could readily be detected after 30 min infection, from both intracellular and extracellular bacteria, however in single copy only very dim fluorescence could be seen. Using Tomato, however, enhanced red fluorescence and was readily visible compared to the DsRed counterpart even in single copy. Overall, the *cysZK* promoter appears to provide very high, constitutive induction of fluorescence in multiple environments. Together, we have not only demonstrated the use of diaminopimelic acid as a flexible selection system for *in vitro* and *in vivo* studies of *Yersinia pestis*, but we have developed reagents that facilitate pathogenesis research using state-of-the-art technology.

## Discussion

Research on highly pathogenic organisms such as *Yersinia pestis* has inherent limitations because of biosafety precautions and those required of genetic engineering. In particular, selection of recombinant DNA, either for retention of exogenous plasmids or to identify recombination events must be restricted to avoid the creation of antibiotic resistant strains that could compromise human treatment options. In this work we sought to establish a system for recombinant DNA expression in the highly pathogenic bacterium *Yersinia pestis* based on metabolic rather than antibiotic selection. Our system targets the biosynthesis of the cell wall, similar to commonly used antibiotics that are effective against *Y. pestis*. Introduction of a null mutation in the *dapAX* operon caused growth dependence on diaminopimelic acid (DAP) for assembly of a functional cell wall. The resulting strain was highly attenuated for virulence in mouse models, and predictably will be in all mammalian hosts as well as the flea vector, none of which harbor pools of DAP. Importantly, no spontaneous reversion to DAP-independent growth was seen either *in vitro* or *in vivo*, thereby greatly increasing the safety associated with *Y. pestis* research.

Unfortunately, the DAP selection system requires working in a mutant strain background which precludes its use on pre-existing strains. However, the benefits of switching to this approach are not limited to the ability to conduct experiments in a safer genetic background. Antibiotic selection in the mammalian or vector host is at best cumbersome, with a requirement for daily or more administration of drug, which may impact the outcome of infection. This introduces experimental risk, including safety concerns, reproducibility of dosing and other, perhaps unpredicted effects on the bacterium or host causing inherent variability and complications with interpretation. Thus, metabolic selection is superior to the introduction of antibiotic resistance for experimental models of infectious diseases.

The DAP system permits *in vivo* selection of plasmids, enabling the faithful study of gene expression by multi-copy plasmids, which has previously not been achieved for *Y. pestis*. To facilitate these studies, we generated *dapAX* mutant strains in multiple *Y. pestis* backgrounds for use in all *in vivo* model systems, including both mammals and fleas (Table S3). Moreover, in this work, we found that both genes in the *dapAX* operon contributed to virulence of *Y. pestis* in mouse models of bubonic and septicemic plague, thereby reducing the potential for spontaneous reversion of virulence. Because of its specific role in virulence, we were able to demonstrate complementation of lethality by expressing *dapX in trans*, showing proof of concept for our system.

We reported the identification of a strong, likely constitutively active *Y. pestis* promoter, with similar activity in *E. coli*, that can drive detectable expression of a fluorescent reporter protein in laboratory media or during macrophage infection. *cysZ* is a conserved, non-essential gene that encodes an inner membrane protein involved in sulfate transport [35], [36]. It is not surprising that sulfate transporter proteins would be highly abundant as this is a key nutrient for cells during all phases of growth. Other metabolite transporter genes have been used in expression vector systems, such as the *lac* operon. Though we and others have employed *lac* promoter constructs for high level expression of recombinant protein in *Y. pestis*, these promoter systems have not been strong enough for single copy use (Eisele, Keleher and Anderson, unpublished observations). Our screen identified optimized production of DsRed and tdTomato under conditions that minimized any impact to bacterial growth. Moreover, because *cysZ* is conserved in other Gram negative bacteria, it is likely that this technology may be broadly useful for pathogenesis research.

## Supporting Information

Table S1 *E.coli* strains and plasmids used in this study.
(DOCX)

Table S2 Primers used for plasmid construction.
(DOCX)

Table S3 Available strains and plasmids not utilized in this manuscript.
(DOCX)
