# Supplementary material for: Novel Genetic Tools for Diaminopimelic Acid Selection in Virulence Studies of Yersinia pestis
Source: PLoS One. 2011 Mar 2;6(3):e17352. doi: 10.1371/journal.pone.0017352 (PMC3047566; doi:10.1371/journal.pone.0017352)
Supplement: Table S1 — E.coli strains and plasmids used in this study. (DOCX) [file pone.0017352.s001.docx]

Table S1. *E.coli* strains and plasmids used in this study.

| **Strain/Plasmid** | **Key Properties** | **Reference** |
| --- | --- | --- |
| ***E.Coli* Strains** |  |  |
| S17-1 | Sm^r^ Sp^r^ Tra^+^; *pro thi hsdR recA*; chromosomal integration of RP4-2-Tc::Mu-*Kan*::Tn*7* λ*pir*+ | [21] |
| JM109 | *recA*1 *endA*1 *gyrA*96 *thi* *hsdR*17 *supE*44 *relA*1 Δ(*lac-proAB*)/F' [*traD*36, *proAB*^+^, *lacI*^q^, *lacZ*ΔM15 | [20] |
| DH5α | F^-^ , *lacZ*ΔαM15 *endA1 recA1 hsdR17*(r_M_- m_K_-) *supE44 thi-1 gyrA96* Δ(*lacZYA-argF*)U169 | [19] |
| **Plasmids** |  |  |
| pCVD442 | Ap^r^, R6K *Ori* and *sacB* counter selection; suicide vector | [22] |
| pCVD442-*dapAX* | Used to delete the *dapAX* promoter and *dapA* open reading frame | This Study |
| pACYC177 | Cloning vector; Ap^r^ Kan^r^, p15a origin of replication | New England Biolabs |
| pBR322 | Cloning vector Ap^r^ Kan^r^, ColE1 origin of replication | New England Biolabs |
| pACYC-177*dapA* | Ap^r^, expresses *dapA* from the endogenous promoter; *dapA* inserted in place of Kan^r^ gene. | This Study |
| pUC18R6KT  Mini-Tn7 | Ap^r^, R6K *Ori* ; mini-Tn7 transposition suicide vector, allows for single copy chromosomal insertion at *att*Tn7 sites. | Choi, 2005 |
| pTNS2 | Ap^r^, R6K *Ori* ;mini-Tn7-T helper plasmid, encodes TnsABC +D transposase complex to catalyze high frequency insertion | Choi, 2005 |
| pDB1 | pUC18R6KT; Ap^r^, *dapA* inserted between att-Tn7 sites | This Study |
| pDB2 | pUC18R6KT; Ap^r^, *dapAX* inserted between att-Tn7 sites | This Study |
| pDsRed-monomer | Ap^r^, *lac* driven DsRed expression | Clontech |
| ptdTomato | Ap^r^, *lac* driven Tomato expression | Clontech |
| pRsaI-2.1 | Ap^r^, *cysZK* driven DsRed expression | This Study |
| pNE160 | Ap^r^, *cysZK* driven Tomato expression | This Study |
| pLK52 | Ap^r^, pDB2 derivative with *cysZK* DsRed cassette from pRsaI-2.1 and *dapAX* between att-Tn7 sites | This Study |
| pNE168 | Ap^r^, pDB2 derivative with *cysZK* Tomato cassette from pNE160 and *dapAX* between att-Tn7 sites | This Study |
| pGEM-T easy | Ap^r^, blue/white screen cloning vector | Promega |
