# Supplementary material for: Novel Genetic Tools for Diaminopimelic Acid Selection in Virulence Studies of Yersinia pestis
Source: PLoS One. 2011 Mar 2;6(3):e17352. doi: 10.1371/journal.pone.0017352 (PMC3047566; doi:10.1371/journal.pone.0017352)
Supplement: Table S2 — Primers used for plasmid construction. (DOCX) [file pone.0017352.s002.docx]

Table S2. Primers used for plasmid construction.

| **Name** | **Sequence (5’ – 3’)** | **Plasmid** |
| --- | --- | --- |
| Dap P1 | ACA TGC ATG CAT CTG CGC CTG GGT TA | pCVD442*dapAX* |
| Dap P2 | GGA ATT CTG ATG GCA ATA TCA TTG C | pCVD442*dapAX* |
| Dap P3 | TCC CCC GGG GGA AGA AGT TAC AGC AA | pACYC-177*dapA* |
| Dap P4 | CCC AAG CTT GGG AAA CCT GCT GCC AA | pACYC-177*dapA* |
| Dap P5 | GGA ATT CGA TGG TAA CCC CAA AGA GC | pCVD442*dapAX* |
| Dap P6 | GCT CTA GAG GTG GAT GCC ATT GTA GG | pCVD442*dapAX* |
| F-+150bp *dapA* | TCC CCC GGG TCG CTC TTC CTG TCA TGC TC | pDB1and pDB2 |
| R-+150bp *dapA* | GGA CTA GTT GGC GCT GTA AGA TAA GA | pDB1 |
| R- *dapAX* | GGA CTA GTT TAT TTT ATC GCG GTT GC | pDB2 |
| F- *DsRed* | GGA ATT CCG ATA TCA TGA CCA TGA TTA CGC CAA G | pLK52 and pRsaI-2.1 |
| R- *DsRed* | ATC GGC CGC TAC TGG GAG CCG GAG TGG C | pLK52 and pRsaI-2.1 |
| F- *CyzZK* | AAA GAT ATC ACT CTC GCC AAT ATT ATT GC | pNE160 |
| R- *CysZK* | AAA GAT ATC ACC TAC CCC GGA GAC AAA GA | pNE160 |
| F- *Tomato* | AAA GAT ATC ATG ACC ATG ATT ACG CCA AG | pNE160 and pNE168 |
| R- *Tomato* | AAA CTC GAG TCA CTG CCC GCT TTC CAG TC | pNE160 and pNE168 |
