# Supplementary material for: Novel Genetic Tools for Diaminopimelic Acid Selection in Virulence Studies of Yersinia pestis
Source: PLoS One. 2011 Mar 2;6(3):e17352. doi: 10.1371/journal.pone.0017352 (PMC3047566; doi:10.1371/journal.pone.0017352)
Supplement: Table S3 — Available strains and plasmids not utilized in this manuscript. (DOCX) [file pone.0017352.s003.docx]

Table S3. Available strains and plasmids not utilized in this manuscript.

| **Strain/Plasmid** | **Key Properties** | **Reference** |
| --- | --- | --- |
| ***Y. pestis* Strains** |  |  |
| CO92*ΔdapAX* pCD1- | CO92; Missing *dapA* promoter and entire *dapA* ORF and CD1 virulence plasmid, generated with pCVD442-dapAX | This Study |
| KIM6+ *ΔdapAX* | KIM6+ (Fetherston et al., 1995); Missing *dapA* promoter and entire *dapA* ORF and CD1 virulence plasmid, generated with pCVD442-dapAX | This Study |
| **Plasmid** |  |  |
| pDB3 | Ap^r^, ColE1 origin of replication, expresses *dapAX* from the endogenous promoter; *dapAX* inserted in place of Tet^r^ gene, pBR322 derivative. | This Study  (pBR322 from New England Biolabs) |
